# Supplementary material for: Unraveling the metastasis‐preventing effect of miR‐200c in vitro and in vivo
Source: Mol Oncol. 2024 Oct 15;19(4):1029–53. doi: 10.1002/1878-0261.13712 (PMC11977663; doi:10.1002/1878-0261.13712)

Supplementary Figure S1

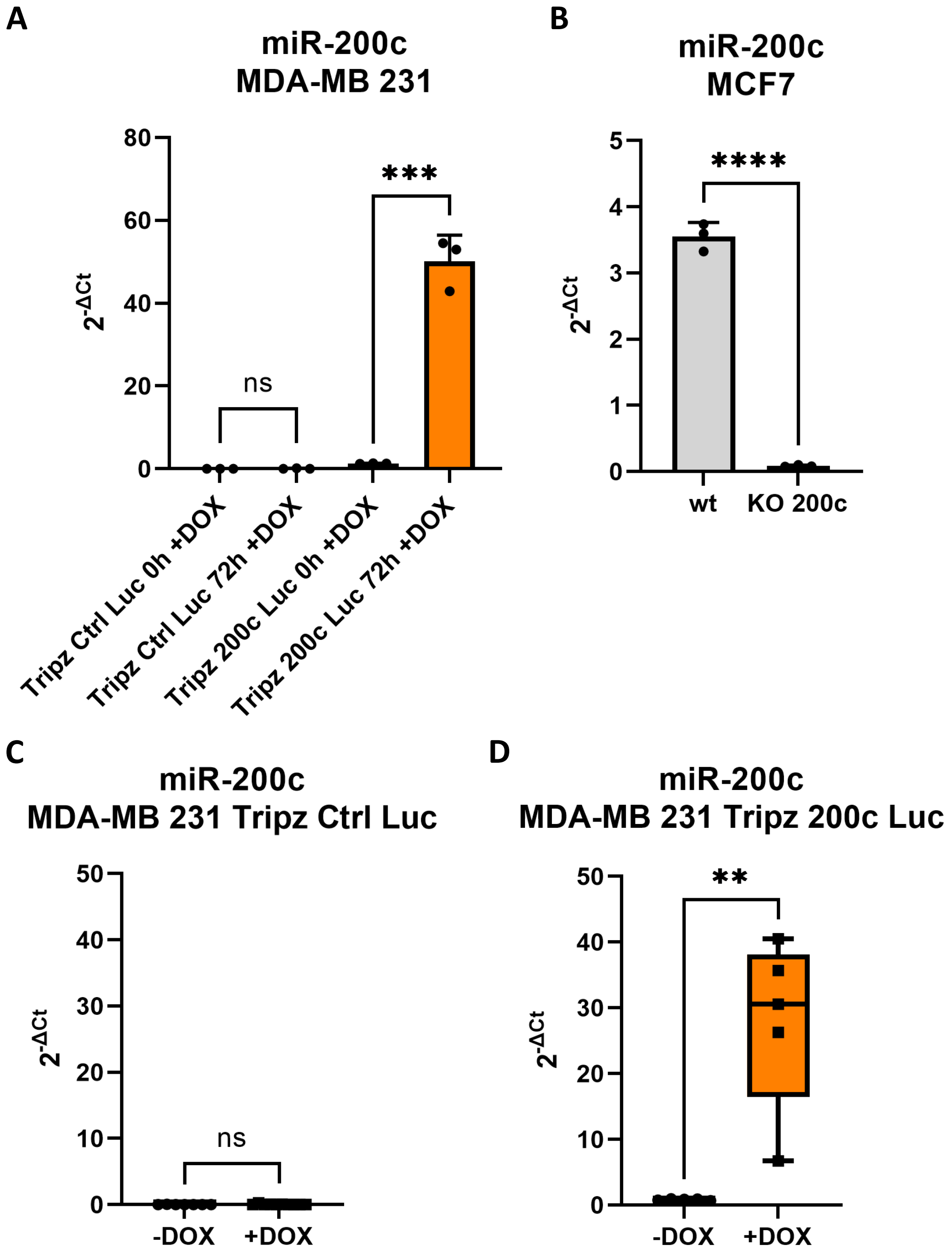

Supplementary Figure S2

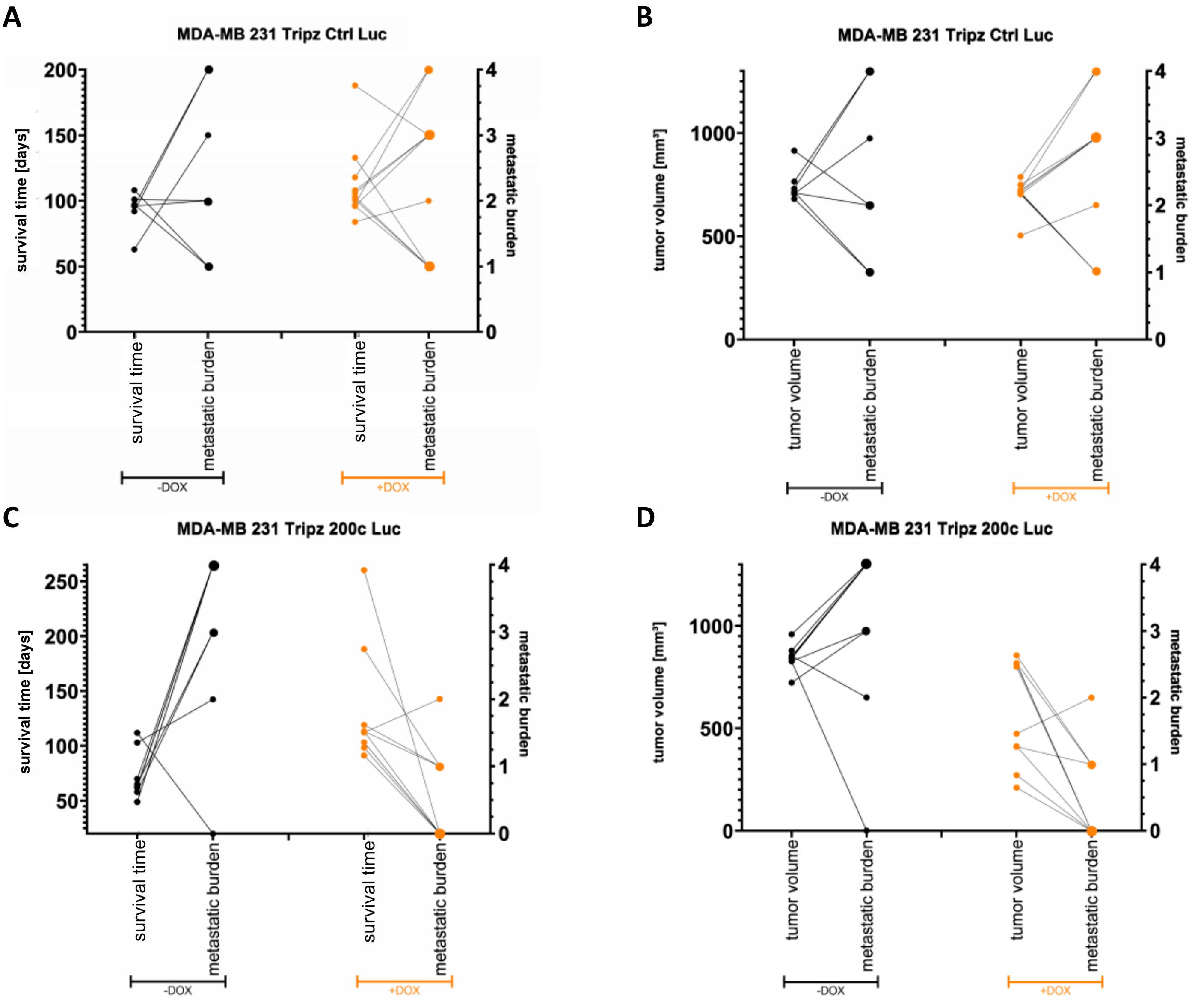

Supplementary Figure S3

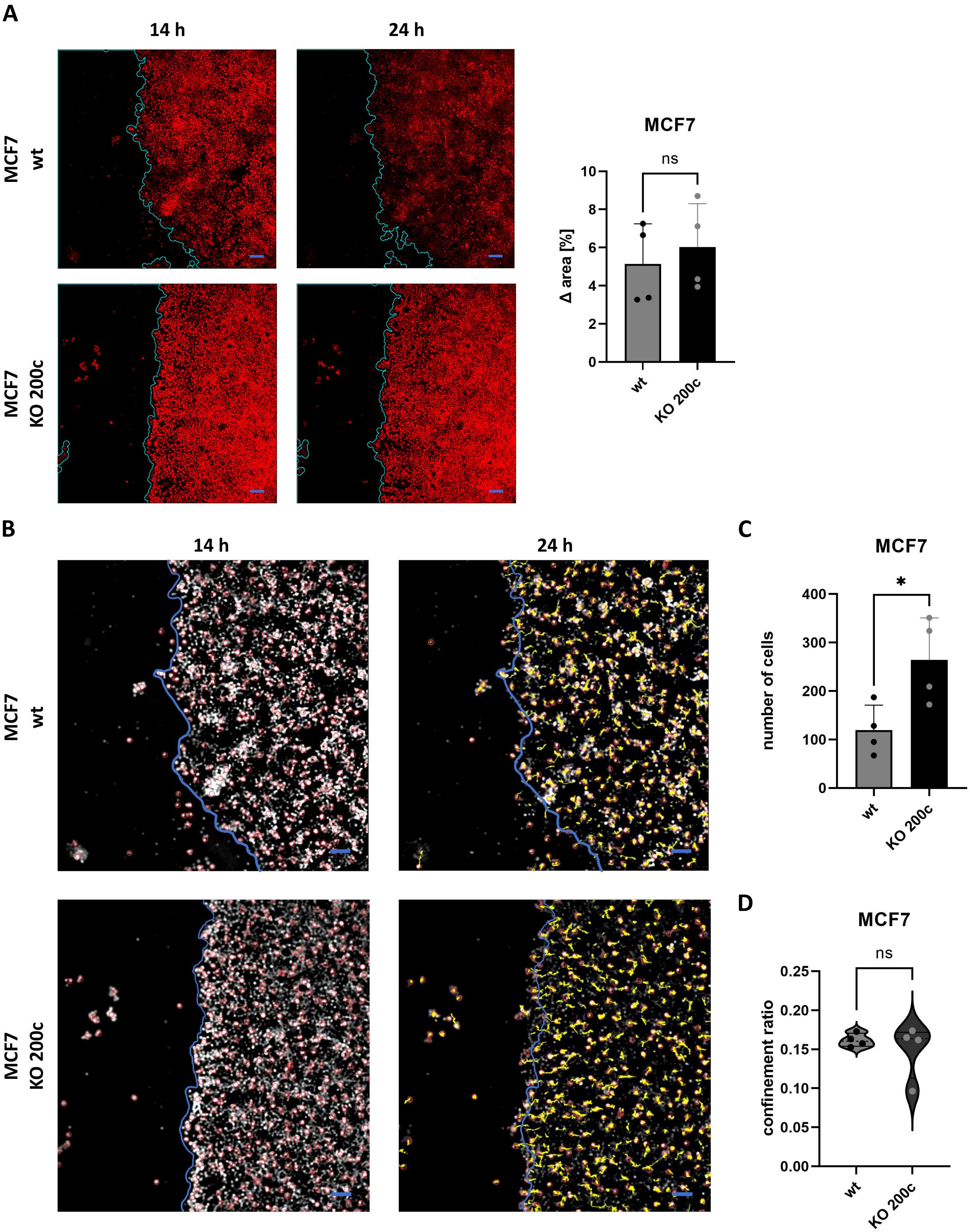

Supplementary Figure S4

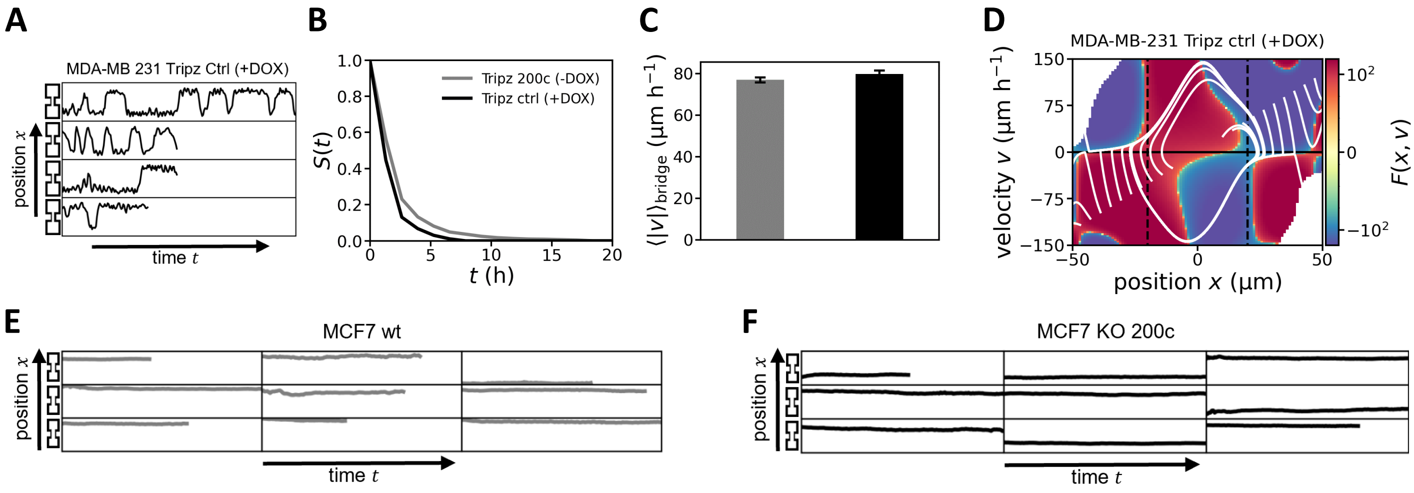

Supplement: Supplementary file 1 — Fig. S1. Expression of miR‐200c is enhanced in MCF7 wildtype (wt) and MDA‐MB 231 Tripz 200c Luc induced with doxycycline (+DOX) cells and tumors, respectively. Validation of miR‐200c expression in vitro and in vivo. (A) Expression level in MDA‐MB 231 Tripz cell systems, at 0 and 72 h after doxycycline induction (n = 3) and in (B) MCF7 wildtype and knockout (KO) miR‐200c (n = 3). Values in (A) and (B) are displayed as mean with SD. For statistical evaluation, an unpaired, two‐tailed Student's t‐test was performed. ns, not significant, ***P < 0.001, ****P < 0.0001. MiR‐200c expression levels in (C) MDA‐MB 231 Tripz Ctrl Luc (n = 7 −DOX and n = 10 +DOX) and (D) MDA‐MB 231 Tripz 200c Luc tumors (n = 5). Data in (C) and (D) are presented as box and whisker plots with minimal to maximal values including all data points. The median is plotted with a line. For statistical evaluation, an unpaired, two‐tailed Student's t‐test was performed. ns, not significant, **P < 0.01. Fig. S2. Reduced metastatic burden in mice with miR‐200c expressing tumor. Presentation of (A) survival time compared to the metastatic burden and (B) tumor volume compared to the metastatic burden of every individual control mouse. Results are separated into normal (black dots, n = 7) or doxycycline‐containing feed (orange dots, n = 11) group. Evaluation of (C) survival time compared to the metastatic burden and (D) tumor volume compared to the metastatic burden of every individual mouse with (orange) or without (gray) miR‐200c expression of the primary tumor. Results are separated into normal (black dots, n = 10) or doxycycline‐containing feed (orange dots, n = 10) group. The bigger the dots the more frequently the same value of the metastatic burden was achieved. Fig. S3. MCF7 breast cancer cells lacking miR‐200c expression tend to leave cell clusters more frequently. (A) Wound closure of MCF7 cells with (wildtype, wt) or without (knockout, KO 200c) miR‐200c expression. Blue lines in the microscopic pict [file MOL2-19-1029-s008.pdf]
